# Concomitant fulvestrant with reirradiation for unresectable locoregional recurrent estrogen receptor positive (ER+) breast cancer: A case report and narrative review

**DOI:** 10.1097/MD.0000000000021344

**Published:** 2020-07-24

**Authors:** Jingxian Ding, Yonghong Guo, Xiaoliu Jiang, Kai Li, Wenbing Fu, Yali Cao

**Affiliations:** a Department of Radiation Oncology, The Third Hospital of Nanchang; b Department of Radiation Oncology, The Fourth Affiliated Hospital of Nanchang University; c Department of Breast Surgery, The Third Hospital of Nanchang, Nanchang, China.

**Keywords:** breast cancer, estrogen receptor positive, fulvestrant, reirradiation, unresectable locoregional recurrence

## Abstract

**Rationale:**

Locoregional recurrence of breast cancer is a challenging issue for clinicians. Treatment options for unresectable recurrent estrogen receptor positive (ER+) breast cancer in previously irradiated area are limited. Some studies showed concomitant fulvestrant with radiation therapy might increase radiosensitivity compared with radiation alone in vitro, no in vivo reports yet.

**Patient concern:**

Here, we present a case report and make a narrative review of concomitant fulvestrant with radiation therapy for unresectable locoregional recurrent ER+ breast cancer. The patient was treated with modified radical mastectomy in 2015, adjuvant chemotherapy, radiotherapy, followed by exemestane until November 2018, relapsed in internal mammary lymph nodes with sternum involved.

**Diagnosis:**

The final diagnosis was breast cancer internal mammary lymph nodes metastasis with sternum involved.

**Interventions:**

After diagnosis was made, concurrent fulvestrant with reirradiation as a palliative treatment were proposed under multiple disciplinary team.

**Outcomes:**

There was a good clinical response, enabling curative chance with radiation therapy to a total dose of 60 Gy. Computed tomography scan revealed no evidence of residual tumor.

**Lessons:**

As far as we know, this is the first report concerning concomitant fulvestrant with reirradiation for unresectable locoregional recurrent ER+ breast cancer. Since no severe adverse events were observed, this strategy could be a suitable “loco-regional rescue therapy” to further reduce tumor progression or even reach a curative effect. Studies of this treatment strategy in randomized clinical trials are warranted to further assess its safety and effectiveness.

## 1 Introduction

Breast cancer is one of the most commonly diagnosed cancers and the leading cause of cancer death among women worldwide. Since the policy of breast cancer screening and advances in adjuvant local and systemic treatment, both recurrence and mortality rates of breast cancer have decreased steadily and 5 year overall survival currently exceeds 90% in some Western countries.^[1,2]^ With a gradual improvement of primary breast cancer treatment, including more extensive use of radiotherapy, improved local disease control over the time has been achieved. However, because of the incidence of breast cancer rising and mortality rates declining, there are an increasing number of breast cancer survivors. Patients with loco-regional recurrence in previously irradiated fields are not uncommon. Locoregional recurrence of breast cancer is a well known independent prognostic factor for distant metastasis and poor survival. Treatment for locoregional recurrence of breast cancer has evolved during the last decades and a multidisciplinary approach is presently strongly considered. Whenever possible, it should be treated with curative intent, preferably with complete excision, otherwise, with biopsy and local and/or systemic therapy as indicated.^[3]^ Unresectable locoregional recurrent breast cancer in previously irradiated area is a life threatening disease and optimal treatment is still a matter of debate. Reirradiation may be a valid treatment option for selected cases.^[4–6]^ Endocrine treatment should be offered to all patients with estrogen receptor positive (ER+) breast cancers. However, in tumors recurring while adjuvant hormonal treatment is ongoing, the benefit of endocrine therapy after relapse is questionable. Fulvestrant is a pure ER antagonist approved for the treatment of metastatic ER+ breast cancer in patients with disease progression following antiestrogen therapy. Studies have demonstrated that fulvestrant radiosensitizes ER+ human breast cancer cells.^[7–10]^ Palliative systemic therapy remains the mainstay of the treatment when local therapy with curative intent is not feasible. Here, we present a successful case of unresectable internal mammary lymph nodes recurrence and sternum involved ER+ breast cancer treated with a concurrent fulvestrant and reirradiation, and make a narrative review of present publications.

## 2 Case report

**Figure 1:**
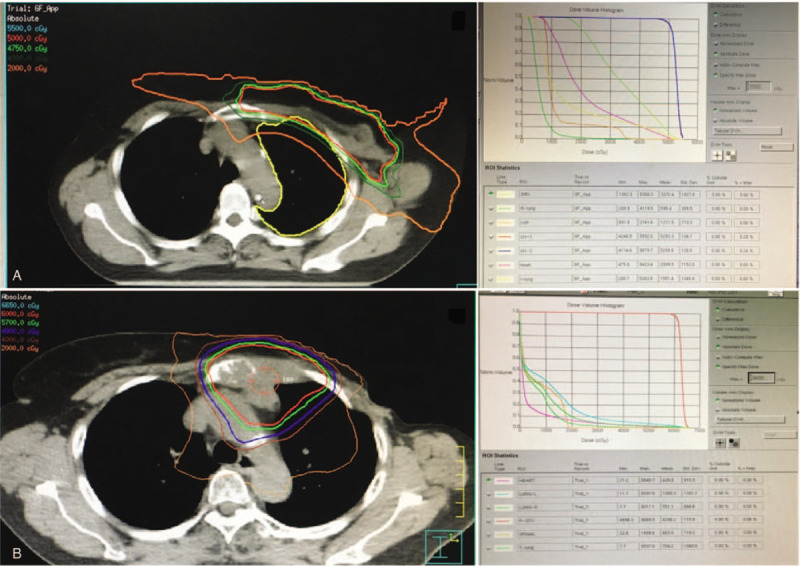
Treatment plan of external beam therapy with isodose distributions and dose distribution-based dose-volume histogram (DVH) of the target volumes and organs at risk. (A) The first course of radiation therapy: top left, representative axial images showing isodose lines and relative chest wall; top right, dose-volume histogram for treatment targets and normal tissues. (B) The second course of reirradiation therapy: bottom left, representative axial images showing isodose lines and relative internal mammary lymph nodes (IMN); bottom right, dose-volume histogram for treatment targets and normal tissues.

**Figure 2:**
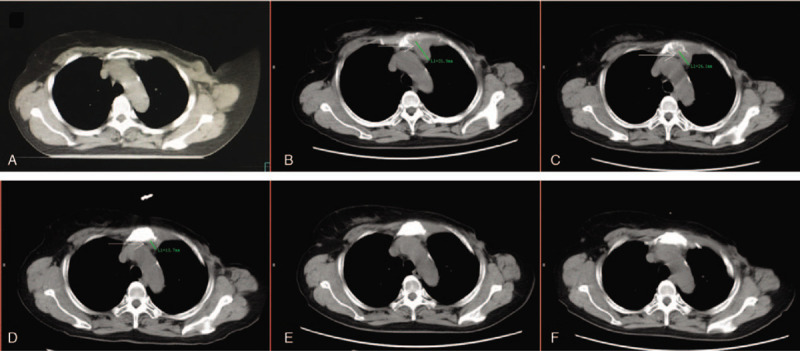
Continuous shrinkage of the internal mammary lymph nodes (IMN) metastases and the involved bone repair, which are marked with arrows. (A) In planning computed tomography (CT) prior to the first course of radiation therapy. (B) CT prior to the second course of reirradiation therapy. (C) In control CT at 21st fraction of reirradiation. (D) Follow-up CT at 1 month after reirradiation therapy. (E) Follow-up CT at 3 months after reirradiation therapy. (F) Follow-up CT at 6 months after reirradiation therapy.

A 59-year-old menopausal woman presented with a complaint of a breast mass, located in the left upper outer quadrant. Family history was negative for both breast and ovarian cancers. On physical examination, there was a palpable, non-tender mass measuring about 4.0 cm in the upper outer quadrant of the left breast. There was no nipple discharge, but it was possible to identify skin retraction around the mass. Axillary lymph nodes were palpable and movable. The rest of the physical examination was within normal limits. Mammography showed a high-density mass without calcification and an indistinct margin in the upper outer quadrant of the left breast. Ultrasonography revealed a 4.3 cm sized hipoecogenic mass in the same location. The core needle biopsy revealed invasive ductal carcinoma (IDC). The clinical stage was determined to be cT2N1M0 according to the 2017 AJCC staging system. The patient had modified radical mastectomy in August 2015. The histological diagnosis was IDC, grade III, measuring 3.5 × 3.5 × 2.5 cm. 23 lymph nodes were excised, 18 of which with metastasis. Immunohistochemical (IHC) examination showed the presence of ER in 60% of tumor cells with weak staining, while progesterone receptors (PR) and HER-2 showed negative, Ki67 was positive in 70% tumor cells. The pathologic stage was determined to be pT2N3 (Stage IIIc). The patient received postoperative adjuvant chemotherapy consisting of 4 cycles of epirubicin and cyclophosphamide, then 12 cycles of weekly paclitaxel followed between August 2015 and February 2016. She also received postoperative adjuvant radiotherapy (RT), with total dose of 50 Gy at 2 Gy/day × 25 fractions to the chest wall and the supra-/infra-clavicular region (SCN), with intensity modulated radiation therapy (IMRT) and 6 MV energy. RT was completed on March 2016. Dose of organs at risk (OARs) in the first course of radiation therapy referred to Table 1. To complete the treatment, the patient received hormonal therapy daily, with exemestane, from March 2016 to November 2018. The patient was kept in follow-up, and there was no evidence of locoregional or distant recurrence for about 2 years. On November 2018, the manubrium pain was noticed. The rest of the physical examination was still within the normal limits. Bone scan indicated sternal metastasis, chest computed tomography (CT) scan revealed the internal mammary lymph nodes (IMN) enlargement, and the core needle biopsy revealed invasive ductal carcinoma (IDC), IHC showed ER+ in 30% of tumor cells with weak staining, while PR and HER-2 showed negative, Ki67 was positive in 90% tumor cells. The patient underwent adequate examination which did not reveal any other distant disease. Concomitant fulvestrant with irradiation was prescribed under our multiple disciplinary team (MDT) from December 17, 2019. Fulvestrant 500 mg intramuscular injection, on days 0, 14, 28, then every 28 days, the fractionation scheme is 60 Gy in 30 fractions over 6 weeks. Treatment plan of external beam therapy with isodose distributions and dose distribution-based dose-volume histogram (DVH) of the target volumes and organs at risk referred to Figure 1. Dose of OARs in the second course of reirradiation therapy referred to Table 2. After completion of irradiation, no severe treatment related adverse events were monitored, and fulvestrant and zoledronic acid were applied monthly. Chest CT scan was routinely applied every month, and the enlarged internal mammary lymph nodes shrank and the involved sternum repaired gradually (Fig. 2). The response staging was clinically complete response according to RECIST 1.1 (Response Evaluation Criteria in Solid Tumors). There was no progression of disease detected to date.

**Table 1:**
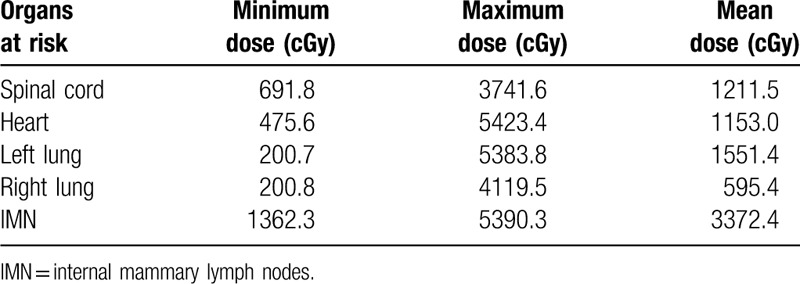
Dose of organs at risk in the first course of radiation therapy.

**Table 2:**
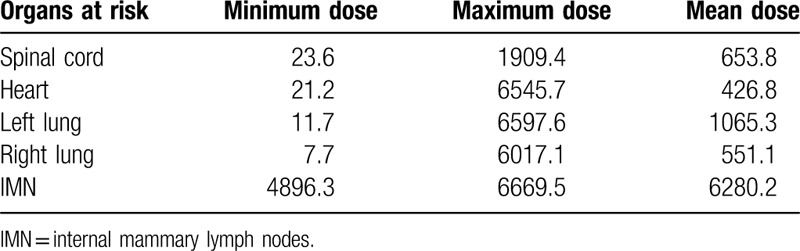
Dose of organs at risk in the second course of reirradiation therapy.

## 3 Discussion

Breast cancer is the most commonly diagnosed tumor and the second leading cause of cancer death in women.^[1,2]^ Currently, breast cancer is treated with a multidisciplinary approach comprising surgery, radiation therapy, chemotherapy, and/or endocrine therapy. This combination is related to a reduction in breast cancer mortality and an accumulation of breast cancer survivors. As the survival time extending, ipsilateral breast tumor recurrence in previous radiation field is not uncommon.^[11]^ Concerning the management of locoregional breast cancer recurrence, the treatment is not standardized and demands a multidisciplinary approach. The management of cancer relapse in previously irradiated field is a challenging therapeutic problem where the balance between quality of life and local control has to be weighed against the side effects of reirradiation therapy.

Treatment options for in-field relapse include surgery, chemotherapy and reirradiation. Local excision alone for chest wall tumor relapse results in poor local control, with high recurrence rates.^[12]^ Reirradiation may reduce the risk of local failure.^[5,13,14]^ Considerations regarding reirradiation include the initial treatment delivered, the time interval to relapse. The external-beam irradiation is a well-documented option for irradiation. However, the clinical indications for reirradiation, radiation therapy techniques, ideal doses and fractionation schemes are far from clearly defined. Unresectable tumors may be treated with concomitant chemotherapy and reirradiation, especially for hormone independent breast cancer. Current clinical practice guidelines recommend radiation be delivered after adjuvant chemotherapy, but the optimal sequencing with adjuvant hormonal therapy has only been partially determined.^[15,16]^ Two clinical studies suggest a detrimental interaction of concomitant tamoxifen and chemotherapy, with sequential treatment having significantly improved disease-free survival and a trend towards improved survival compared to concomitant treatment.^[17]^ Wang et al found the effects of Fulvestrant blocking estrogen at the receptor level and inhibiting estrogen-stimulated cell division in vitro. The cell cycle effects of fulvestrant increased the proportion of cells in G1 arrest, accompanied by a simultaneous decrease in cells in the S phase. Given that cancer cells are most radiosensitive in the G2/M phase, less sensitive in G0/G1, and least sensitive in the latter part of the S phase, and cell cycle progression can be stopped at the G1, S, and G2 checkpoints, they concluded that the decreased proportion of cells in S phase before irradiation may result in the observed decrease in the surviving fraction with Fulvestrant + RT. Furthermore, the increased proportion of cells arrested in the G2 phase in Fulvestrant + RT treatment group may also contribute to the decrease of the surviving fraction.^[7,18]^ The optimal sequencing of radiotherapy with endocrine therapy remains to be determined.

Fulvestrant has been found to be as effective as the third-generation aromatase inhibitors (AIs) anastrozole and letrozole for advanced, post-menopausal, tamoxifen-resistant breast cancer, and also appears to be effective after treatment with non-steroidal AIs.^[19]^ Based on the evidence available, for the patient we presented here, concomitant fulvestrant with irradiation was prescribed under our MDT. Throughout radiation treatment, patients were examined weekly by the radiation oncologist for acute toxicities using the Radiation Therapy Oncology Group (RTOG) radiation morbidity scoring criteria. The patient demonstrates acceptable acute and late side effects, there were no such grade 4 toxicities appeared. Toxicity is generally well tolerated. 3 months after the completion of reirradiation, the enlarged internal mammary lymph nodes shrank and the involved sternum repaired gradually. This case report demonstrates that ER+ breast cancer patients even with “in field” locoregional recurrence can benefit from simultaneous treatment of fulvestrant and reirradiation. The treatment response, radiation induced adverse events, cosmetic result and quality of life are satisfactory and encouraging. From this case, it seems that locoregionally disease even in the setting of previously radiated field, concomitant fulvestrant with reirradiation may be considered as an option.

## 4 Conclusion

Concomitant fulvestrant with reirradiation seems to be a safe and effective therapy for locoregionally recurrent ER+ breast cancer and improves the outcome of gross tumor disease. Since gross residual disease represents an essential factor for locoregional control and survival outcome, further comprehensive investigations into the simultaneous use of radiosensitizers, such as fulvestrant, with radiotherapy are wanted in randomized clinical trials.

## Acknowledgments

We thank all participants for their supports and participation.

## Author contributions

**Conceptualization:** Jingxian Ding, Yonghong Guo.

**Data curation:** Kai Li, Wenbing Fu

**Project administration:** Yali Cao

**Writing – original draft:** Jingxian Ding.

**Writing – review & editing:** Jingxian Ding, Xiaoliu Jiang, Yali Cao.
